# Exploration of Serum Exosomal LncRNA TBILA and AGAP2-AS1 as Promising Biomarkers for Diagnosis of Non-Small Cell Lung Cancer

**DOI:** 10.7150/ijbs.39123

**Published:** 2020-01-01

**Authors:** Yao Tao, Yuting Tang, Zailin Yang, Futao Wu, Lu Wang, Liyuan Yang, Li Lei, Yipei Jing, Xueke Jiang, Hongjun Jin, Yao Bai, Ling Zhang

**Affiliations:** 1 Key Laboratory of Laboratory Medical Diagnostics Designated by the Ministry of Education, School of Laboratory Medicine, Chongqing Medical University, Chongqing, China.; 2 Department of Clinical Laboratory, The Third Affiliated Hospital of Chongqing Medical University, Chongqing 401120, China; 3 Department of Clinical Laboratory, The First Affiliated Hospital of Chongqing Medical University, Chongqing 400016, China

**Keywords:** Non-small cell lung cancer, Biomarker, Exosomes, Long non-coding RNAs, TBILA, AGAP2-AS1.

## Abstract

Non-small cell lung cancer is the most common type of cancer with a poor prognosis, and development of an effective diagnostic method is urgently needed. Exosomal lncRNAs, a class of transcripts longer than 200 nucleotides packaged into exosomes, have been defined as an ideal diagnostic biomarker for cancer. However, little is known about the clinical utility of exosomal lncRNAs in NSCLC. Here, we aimed to identify exosomal lncRNAs as promising biomarkers for NSCLC diagnosis. First, serum exosomes from NSCLC patients were successfully isolated by a polymer precipitation kit and then identified by TEM, NTA and western blot analysis. A total of nine candidate lncRNAs were detected by qRT-PCR in a training set. The two exosomal lncRNA TBILA and AGAP2-AS1 were screened out for the higher levels in NSCLC patients than that of healthy controls in a validation set. And there was a significant positive correlation between these exosomal lncRNAs levels and tumor size, lymph node metastasis and TNM stage. Additionally, we validated that these exosomal lncRNAs were stable in serum. Next, we evaluated the diagnostic efficiency of exosomal lncRNAs in NSCLC patients by ROC curve analysis. The data showed that individual TBILA or AGAP2-AS1 exhibited better diagnostic efficiency in NSCLC patients with different tumor pathologic subtypes and early stage, whereas the combination of lncRNAs did not provide better results than individual lncRNAs. Notably, the combination of two exosomal lncRNAs and the serum tumor biomarker Cyfra21-1 widely used in clinical practices further improved the diagnostic accuracy for NSCLC patients. This study suggests that exosomal lncRNA TBILA and AGAP2-AS1 may be promising biomarkers for diagnosis of NSCLC.

## Introduction

Lung cancer remains the leading cause of cancer-related mortality worldwide, especially in China 1, 2. Non-small cell lung cancer (NSCLC) is the most common type of lung cancer with two major pathologic subtypes: adenocarcinoma (ADC) and squamous cell carcinoma (SCC) 3. The majority of patients with NSCLC are diagnosed at advanced stage with only 21% of 5-year survival rate, thus the early detection of NSCLC is necessary to reduce disease mortality rates 4. Currently, low-dose computerized tomography (LDCT) scans are the predominant screening methods used for detection of NSCLC. It has well known that LDCT can detect more pulmonary nodules, whereas it may lead to over-diagnosis and unnecessary surgeries due to high false-positive rates 5. In addition, blood-based tests have been developed to complement imaging modalities. However, these serum protein biomarkers of NSCLC currently used in clinical practice, such as cytokeratin 19 fragment (Cyfra21-1), carcinoembryonic antigen (CEA) and SCC antigen, often exhibited lower diagnostic sensitivity and specificity 6. Therefore, a novel biomarker for diagnosis of NSCLC is urgently needed.

Exosomes are a kind of extracellular membranous vesicle with a diameter of approximately 40-100 nm. It has been well acknowledged that exosomes contain proteins, lipids, and nucleic acids, such as DNAs and various forms of RNAs 7. They are often released into the microenvironment and act as vehicles in cell-to-cell communication 8, 9. Great deals of reports have indicated that exosomes-mediated transfer of molecular constituents remodels the tumor microenvironment and contributes to carcinogenesis 10, 11. Notably, exosomes are often found in various human body fluids, including peripheral blood (serum/plasma), urine and saliva 12, 13. Moreover, the exosomal membrane could protect various types of molecules from enzymatic degradation in circulation 14. In recent years, exosomes have been extensively studied in the medical field as a new delivery system and therapeutic targets. More importantly, exosomes are increasingly recognized as a liquid biopsy technique to aid in the diagnosis of malignancies 15. Thus, exosomes that contain some specific nucleic acids and proteins should be exploited as biomarkers in clinical application of NSCLC.

Long non-coding RNAs (lncRNAs) are broadly defined as a class of transcripts longer than 200 nucleotides with limited protein-coding capacity 16. Evidence has revealed that lncRNAs are differentially expressed in cancer tissues and play critical roles in the initiation and progression of tumors 17-20. Interestingly, cancer-derived lncRNAs can be packaged into exosomes and transported in circulating blood, which makes exosomal lncRNAs as an ideal noninvasive diagnostic and prognostic biomarker for cancer detection 21. From recent studies, Lin et al. demonstrated that plasma exosomal lncRNA UEGC1 originating from gastric cancer cells was highly sensitive to the early stage of gastric cancer 22. Moreover, Sedlarikova et al. reported that serum exosomal lncRNA PRINS could act as a diagnostic biomarker in multiple myeloma 23. Another study also declared that serum exosomal lncRNA PCAT-1, UBC and SNHG16 might predict the recurrence of bladder cancer 24. To date, a large number of lncRNAs have been clarified in the tissues of different cancers, however, serum-derived exosomal lncRNAs as diagnostic biomarkers for NSCLC have not been well explored.

In this study, we adopted a multistep, case-control study to explore the clinical value of serum exosomal lncRNAs as biomarkers for diagnosis of NSCLC. Here, we identified the upregulated lncRNAs in serum exosomes from NSCLC patients by two independent sets. Furthermore, we assessed the correlations between exosomal lncRNAs levels and clinical characteristics of NSCLC. Finally, we evaluated the diagnostic efficiency of exosomal lncRNAs by ROC curve analysis. This study suggests that exosomal TBILA (TGFβ-induced lncRNA) and AGAP2-AS1 (AGAP2 antisense RNA 1) may be novel potential biomarkers and the combination of two exosomal lncRNAs and Cyfra21-1 could improve the diagnostic accuracy for NSCLC patients.

## Materials and Methods

### Study subjects

All patients were enrolled from The First Affiliated Hospital of Chongqing Medical University and The Third Affiliated Hospital of Chongqing Medical University from April 2018 to August 2019. All patients were newly diagnosed as NSCLC by histopathology according to the 2015 World Health Organization Classification of Tumors of the Lung 25. Patients treated with chemotherapy or radiotherapy before blood collection were excluded. Health individuals matched for sex and age were recruited as healthy controls. A total of 150 patients with NSCLC, including 86 ADCs and 64 SCCs, and 150 healthy controls were enrolled. All samples were randomly separated into a training set (50 NSCLC cases and 50 controls) and a validation set (100 NSCLC cases and 100 controls). Details of the clinical characteristics are provided in Table 1. In addition, 10 paired serum samples from NSCLC patients before and 1 month after the surgery (7 men, 3 women; mean age 65 years) were collected. All samples were collected with informed consent and this study was approved by the Ethics Committees of Chongqing Medical University.

### Isolation of serum exosomes

Five milliliters of venous blood from each participant was separated into serum and cellular fractions by two-step centrifugation protocol (2,000 × g for 5 min at 4°C; 10,000 × g for 5 min at 4°C). Each serum supernatant was transferred to RNase-free tube for further usage. The isolation of exosomes from serum was performed using the Total Exosome Isolation Reagent (Invitrogen, CA, USA). First, 500 μL serum samples were mixed with 100 μL of exosome precipitation reagent and incubated at room temperature for 10 min. Next, the mixture was centrifugated at 10,000 × g for 10 min. Then, the supernatants were collected and used as exosomes-depleted serum (EDS). Finally, the exosomes-containing pellet was resuspended in 200 μL of phosphate-buffered saline (PBS) for further analysis.

### Identification of serum exosomes

The morphology of exosomes was visualized by the transmission electron microscopy (TEM). The exosomes resuspension was loaded onto a carbon-coated 300 mesh copper grid (ProSciTech, QLD, Australia), followed by drying for 5 min at room temperature. Then, the grid was stained with a drop of 2% phosphotungstic acid (Sigma, CA, USA) for 10 min at room temperature. The morphology of exosomes was visualized by the electron microscope H-7600 (Hitachi, Tokyo, Japan).

The size distribution and concentration of the isolated exosomes were assessed by the nanoparticle tracking analysis (NTA). Exosomes resuspended in PBS were injected into the ZetaView PMX 110 (Particle Metrix, Meerbusch, Germany), and the exosomes particles were tracked and sized based on Brownian motion and the diffusion coefficient. Data analysis was performed with the corresponding software, ZetaView 8.02.28.

The exosomes marker proteins were detected by western blot analysis. Total protein of exosomes and EDS was extracted with RIPA lysis buffer supplemented with protease inhibitor cocktail (Roche, Basel, Switzerland) and quantified with bicinchoninic acid assays (Beyotime, Shanghai, China). Equal loading of extracted protein was separated by SDS-PAGE, followed by being transferred to polyvinylidene difluoride (PVDF) membrane (Thermo Fisher Scientific, CA, USA). The membrane was blocked with 5% non-fat milk for 3 hrs, followed by incubating with diluted CD9, CD63 and Tubulin antibodies (1:1000, Santa Cruz Technologies, CA, USA) overnight at 4°C.

### RNA extraction from exosomes and quantitative real-time PCR (qRT-PCR)

Extraction of RNA from exosomes was performed using TRIzol LS Reagent (Invitrogen, CA, USA). The extracted RNA was transcribed into cDNA using the PrimeScript™RT Master Mix (Takara, Kyoto, Japan). qRT-PCR analysis was performed on CFX ConnectTM real-time system (Bio-Rad, CA, USA) according to the manufacturer's instructions. *GAPDH* was used as a reference gene in qRT-PCR analysis. The relative expression levels of lncRNAs were calculated using the 2^-ΔCT^ method. Details of the primer sequences used in qRT-PCR are shown in Table 2.

### Stability testing of exosomal lncRNAs

Three tests were performed to investigate the stability of exosomal lncRNAs. In the test of distribution of lncRNAs, 10 serum samples were used to separate exosomes-enriched fraction and exosomes-depleted serum (EDS) fraction by the Total Exosome Isolation Reagent (Invitrogen, CA, USA). In the RNase treatment experiment, RNase A (Fermentas, MD, USA) was added to exosomes suspension derived from 20 serum samples at a final concentration of 2 μg/mL and incubated for 20 min at 37°C. In the test of room temperature incubation, exosomes suspension derived from 3 serum samples were placed at room temperature for 0, 12, 24 and 48 hrs. After the above treatments, extraction of RNA from exosomes and quantification of lncRNAs were performed using the aforementioned methods.

### Cyfra21-1 measurement

The concentration of serum Cyfra21-1 was measured by chemiluminescence method using the Roche Cobas e601 (Roche, Basel, Switzerland). The upper limit of reference interval for Cyfra21-1 is 3.3 ng/mL.

### Statistical analysis

All data were derived from three independent experiments. Data were presented as mean ± standard deviation (SD). Clinical characteristics among groups were compared by χ^2^ test. The different expression of lncRNAs among groups was determined using the Mann-Whitney unpaired *t*-test or paired *t*-test. Receiver operating characteristic (ROC) curves analysis was performed to determine the area under the curves (AUC), sensitivity and specificity of exosomal lncRNAs by Medcalc 15.2.2 (MedCalc, Mariakerke, Belgium). The Youden index was used to determine the optimal cut-off value of serum exosomal lncRNAs. Binary logistic regression analysis was used to establish the combination of markers for discriminating NSCLC from healthy controls. All calculations were carried out using SPSS 19.0 (IBM, NY, USA) and GraphPad 7.0 (GraphPad Software, CA, USA). A *p*-value < 0.05 was considered statistically significant.

## Results

### Identification of exosomes in serum

**Figure 1:**
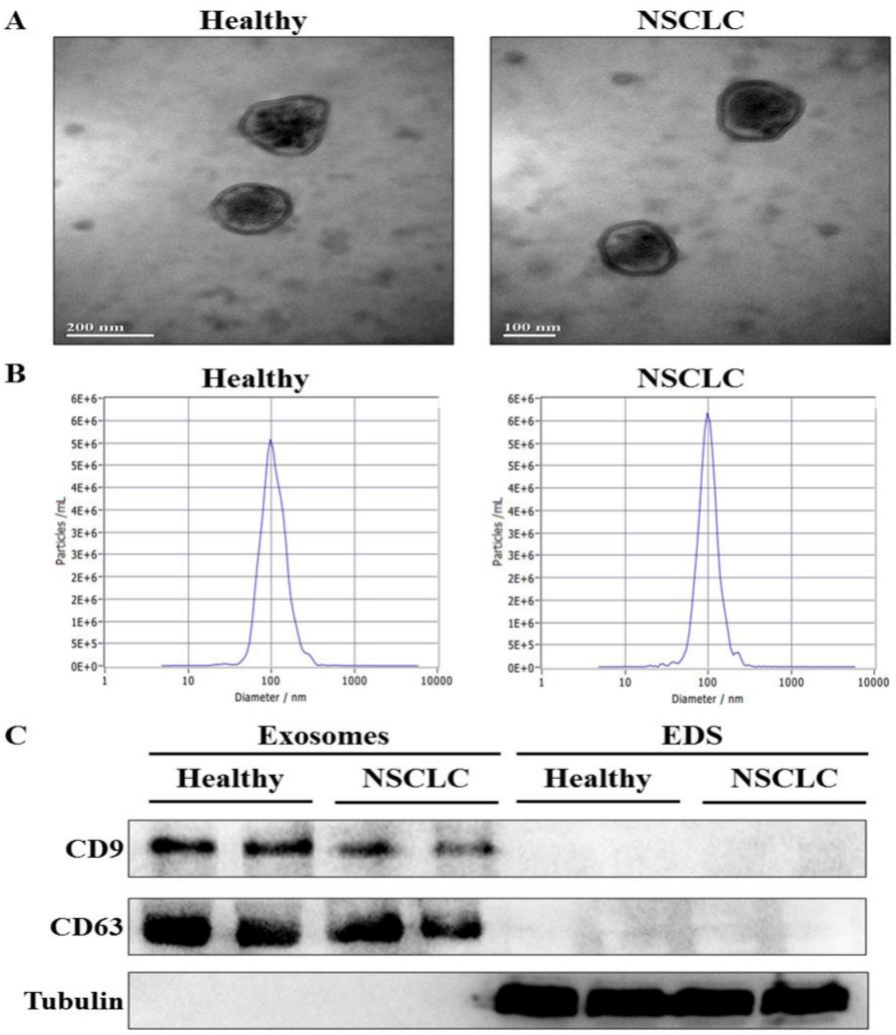
Identification of exosomes in serum.** (A)** The morphology of serum exosomes was visualized by transmission electron microscopy (TEM). **(B)** The size distribution and concentration of serum exosomes were assessed by nanoparticle tracking analysis (NTA). **(C)** The specific exosome marker protein (CD9 and CD63) and non-exosomal protein (Tubulin) in serum exosomes and exosomes-depleted serum (EDS) were detected by western blot analysis.

To confirm whether exosomes were successfully isolated from serum, transmission electron microscopy (TEM) and nanoparticle tracking analysis (NTA) were used to characterize the isolated exosomes. The results showed that the isolated exosomes had double layer membrane structure (Fig. 1A), and the size distribution of exosomes was approximately 100 nm in diameter (Fig. 1B). Moreover, western blot analysis demonstrated the presence of the specific exosome marker protein (CD9 and CD63) in the exosomes-enriched fractions, but not in EDS. On the other hand, tubulin as a negative control was absent in the exosomes-enriched fractions (Fig. 1C). These results indicated that exosomes were isolated from serum successfully.

### Screening of exosomal lncRNAs in a training set

A total of nine lncRNAs, which were differentially expressed in the tissues of NSCLC and played critical roles in carcinogenesis in the latest literature 26-34, were selected as candidates. The levels of serum exosomal lncRNAs were quantitated by qRT-PCR in the training set consisting of 50 NSCLC cases and 50 healthy controls. The results showed that three lncRNAs (TBILA, AGAP2-AS1 and SOX2OT) transcripts were significantly increased in serum-derived exosomes from NSCLC patients compared with that in healthy controls (*p* < 0.001, Table 3). Therefore, the three exosome lncRNAs were screened out for further study.

### Verification of the selected exosomal lncRNAs levels in a validation set

**Figure 2:**
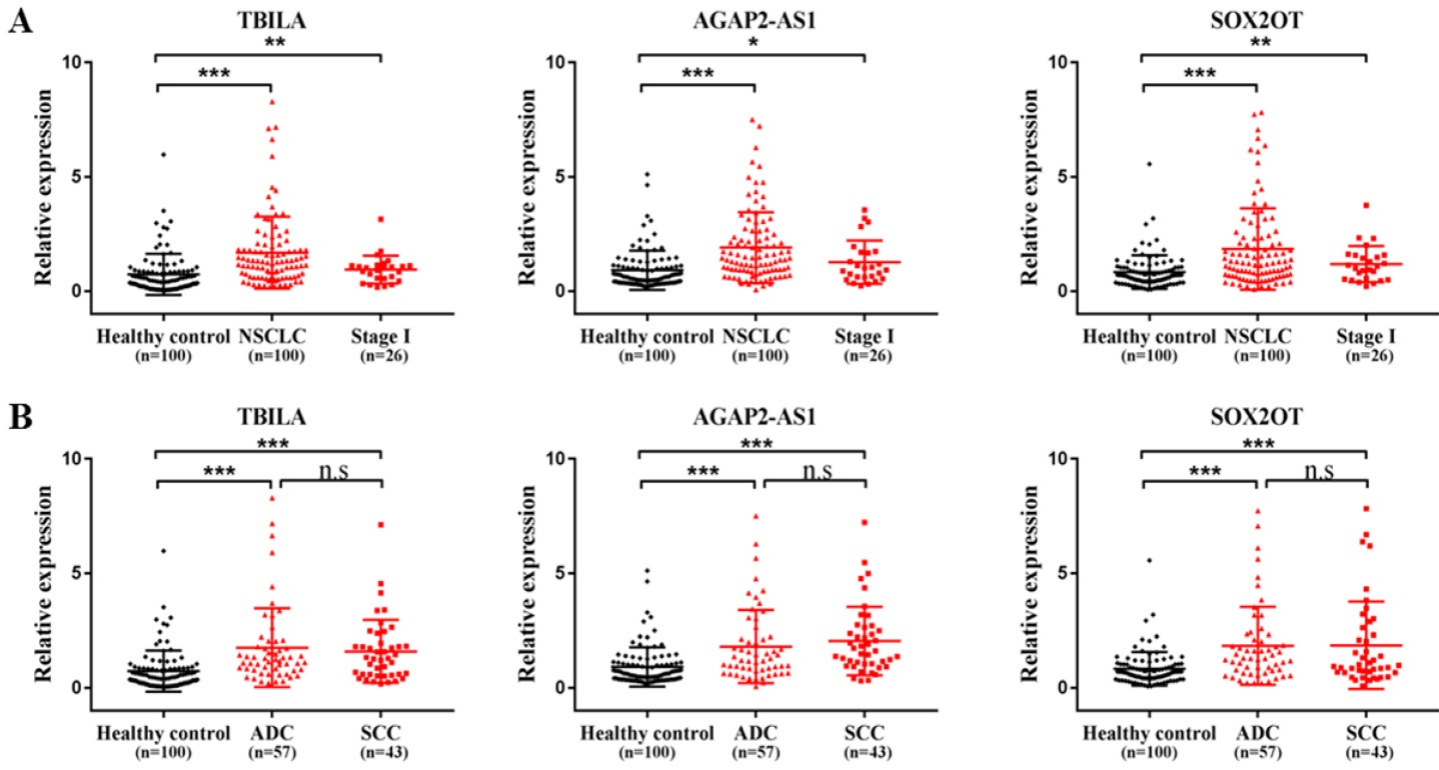
The levels of three exosomal lncRNAs in NSCLC patients in the validation set.** (A)** qRT-PCR analysis of three exosomal lncRNAs in NSCLC patients, stage I NSCLC patients and healthy controls.** (B)** qRT-PCR analysis of three exosomal lncRNAs in lung ADC patients, SCC patients and healthy controls (n=100). * *p* < 0.05, ** *p* < 0.01, *** *p* < 0.001. n.s, no signification, *p* > 0.05.

To further verify the selected three exosomal lncRNAs levels in serum samples from NSCLC patients, we tested the lncRNAs transcripts in the validation set including an additional 100 NSCLC patients and 100 healthy controls. As shown in Fig. 2A, the quantity of three exosomal lncRNAs was significantly increased in patients with NSCLC compared with healthy controls (all, *p* < 0.001). Notably, the elevated levels of three exosomal lncRNAs were also identified in patients with early stage of NSCLC (TBILA and SOX2OT, *p* < 0.01; AGAP2-AS1, *p* < 0.05). In addition, the levels of three exosomal lncRNAs were significantly upregulated in lung ADC patients and lung SCC patients as compared to healthy controls, respectively (all, *p* < 0.001); whereas there was no significant difference in exosomal lncRNAs levels between the two groups (*p* > 0.05, Fig. 2B). Collectively, serum exosomal lncRNA TBILA, AGAP2-AS1 and SOX2OT were higher expression in NSCLC patients than that of healthy subjects.

### Correlation of exosomal lncRNAs levels with clinical characteristics

**Figure 3:**
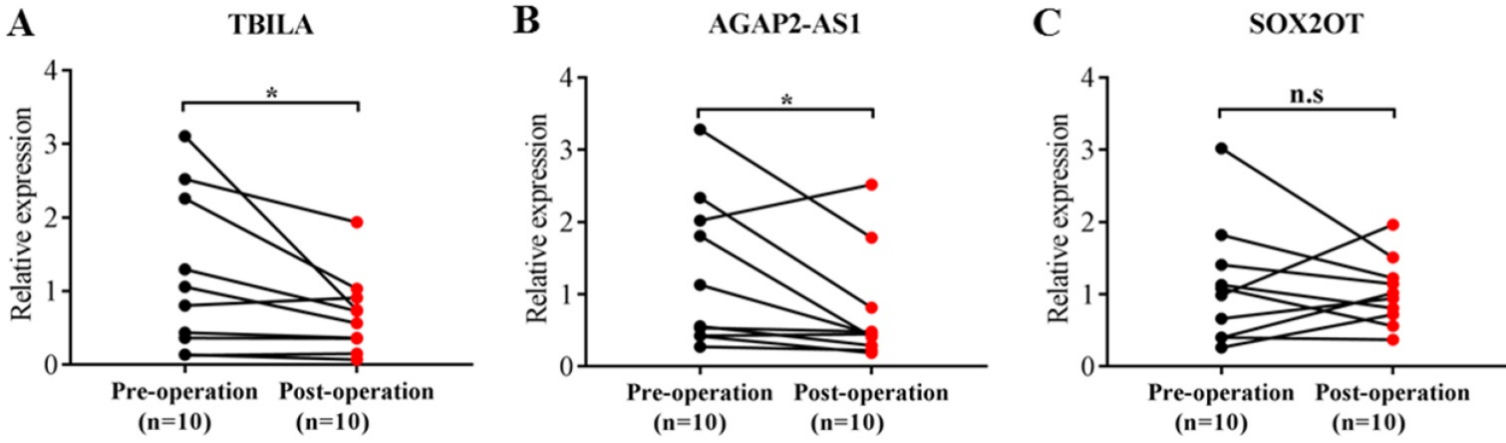
Comparison of three exosomal lncRNAs expression in preoperative and postoperative serum samples of NSCLC patients (n=10).** (A)** TBILA.** (B)** AGAP2-AS1. **(C)** SOX2OT. * *p* < 0.05; n.s, no signification.

To further explore the potential of exosomal lncRNAs as a predictor for NSCLC, we assessed the correlation of three exosomal lncRNAs with clinical characteristics of NSCLC patients. As shown in Table 4, TBILA was significantly correlated with tumor size (*p* < 0.05), while AGAP2-AS1 was significantly correlated with lymph node metastasis and TNM stage (all, *p* < 0.05). However, there was no significant relationship between SOX2OT levels and clinical characteristics (all, *p* > 0.05). In addition, we analyzed the correlation of three exosomal lncRNAs expression and operative status. The results indicated that the levels of TBILA and AGAP2-AS1 were significantly reduced in postoperative samples compared to the paired preoperative samples (all, *p* < 0.05, Fig. 3A-B), whereas there was no statistical difference in SOX2OT levels between the two groups (*p* > 0.05, Fig. 3C). Based on the above experimental results, we focused on exosomal lncRNA TBILA and AGAP2-AS1 for further study.

### Evaluation of the stability of exosomal lncRNAs in serum samples

**Figure 4:**
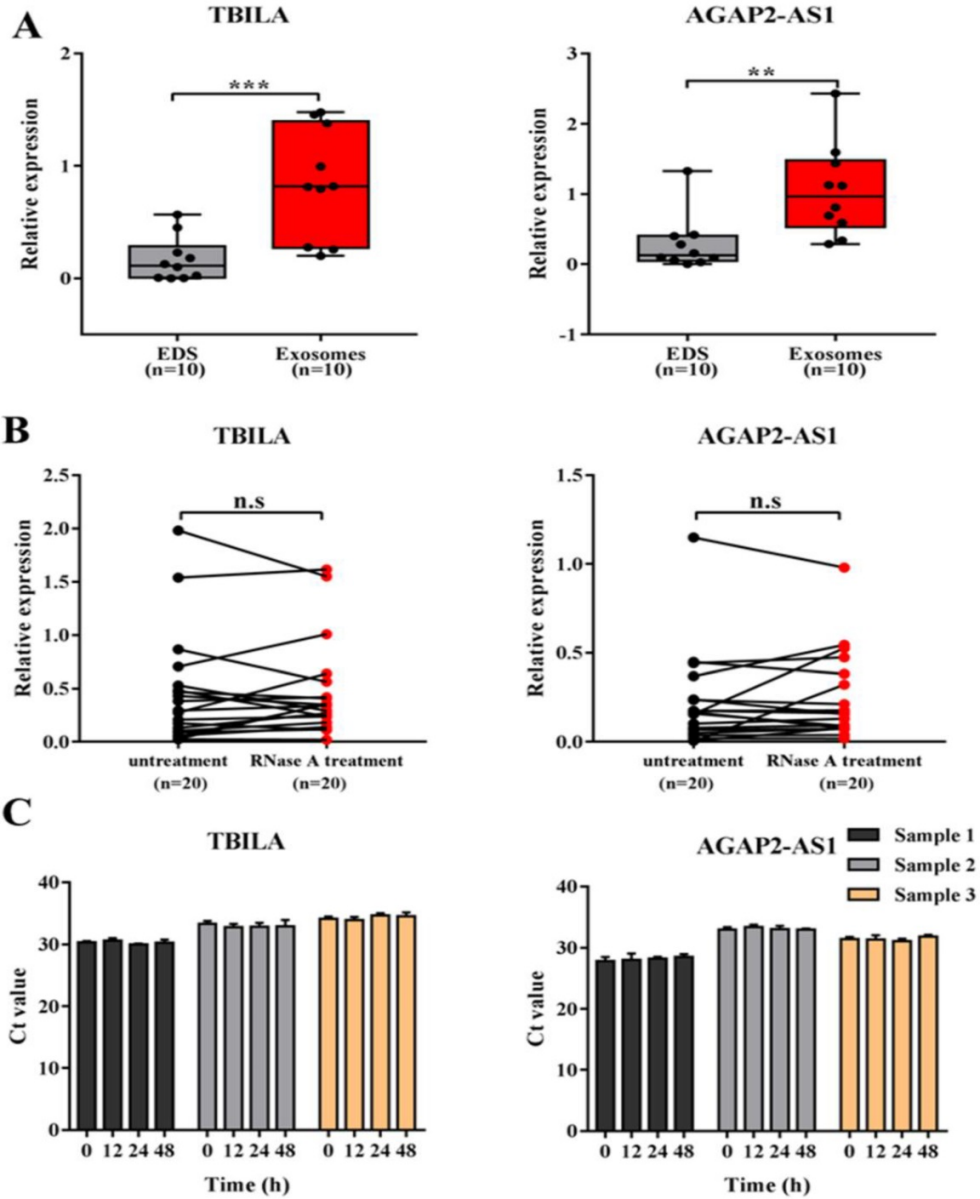
Evaluation of the stability of exosomal lncRNA TBILA and AGAP2-AS1 in serum samples.** (A)** qRT-PCR analysis of lncRNAs levels in serum exosomes and exosomes-depleted serum (EDS) from NSCLC patients **(B)** The exosomes suspensions were treated with or without RNase A (2 μg/mL) for 20 min at 37°C, following by qRT-PCR analysis of lncRNAs. **(C)** The exosomes suspensions were placed at room temperature for different times, following by qRT-PCR analysis of lncRNAs. ** *p* < 0.01; *** *p* < 0.001; n.s, no signification.

Given that better stability is a critical prerequisite for tumor biomarkers, we next evaluated the stability of exosomal lncRNA TBILA and AGAP2-AS1 in serum samples. Firstly, we examined whether the two lncRNAs in serum samples were enriched in exosomes. The levels of TBILA and AGAP2-AS1 in serum exosomes and EDS were detected, respectively. As a result, the levels of two lncRNAs were significantly higher in serum exosomes than that in EDS (TBILA, *p* < 0.001; AGAP2-AS1, *p* < 0.01; Fig. 4A). Next, the exosomes suspensions were directly treated with RNase A and this treatment did not reduce exosomal lncRNAs levels evidently (all, *p* > 0.05; Fig. 4B). Finally, the exosomes suspensions were placed at room temperature for 48 hrs. The data indicated that the levels of these exosomal lncRNAs were not significantly decreased from 0 hrs to 48 hrs at room temperature (Fig. 4C). Collectively, the above results indicated that exosomal lncRNAs had good stability in the serum of NSCLC patients.

### Diagnostic efficiency of exosomal lncRNAs for NSCLC patients

To evaluate the diagnostic efficiency of exosomal lncRNA TBILA and AGAP2-AS1 in NSCLC patients with different subtypes and early stage, ROC curve analysis was plotted to determine the AUC, sensitivity and specificity. In addition, serum tumor biomarker Cyfra21-1 widely used in clinical practices was also included as control. The diagnostic efficiency of them in distinguishing the different status of NSCLC from controls were summarized in Table 5.

**Figure 5:**
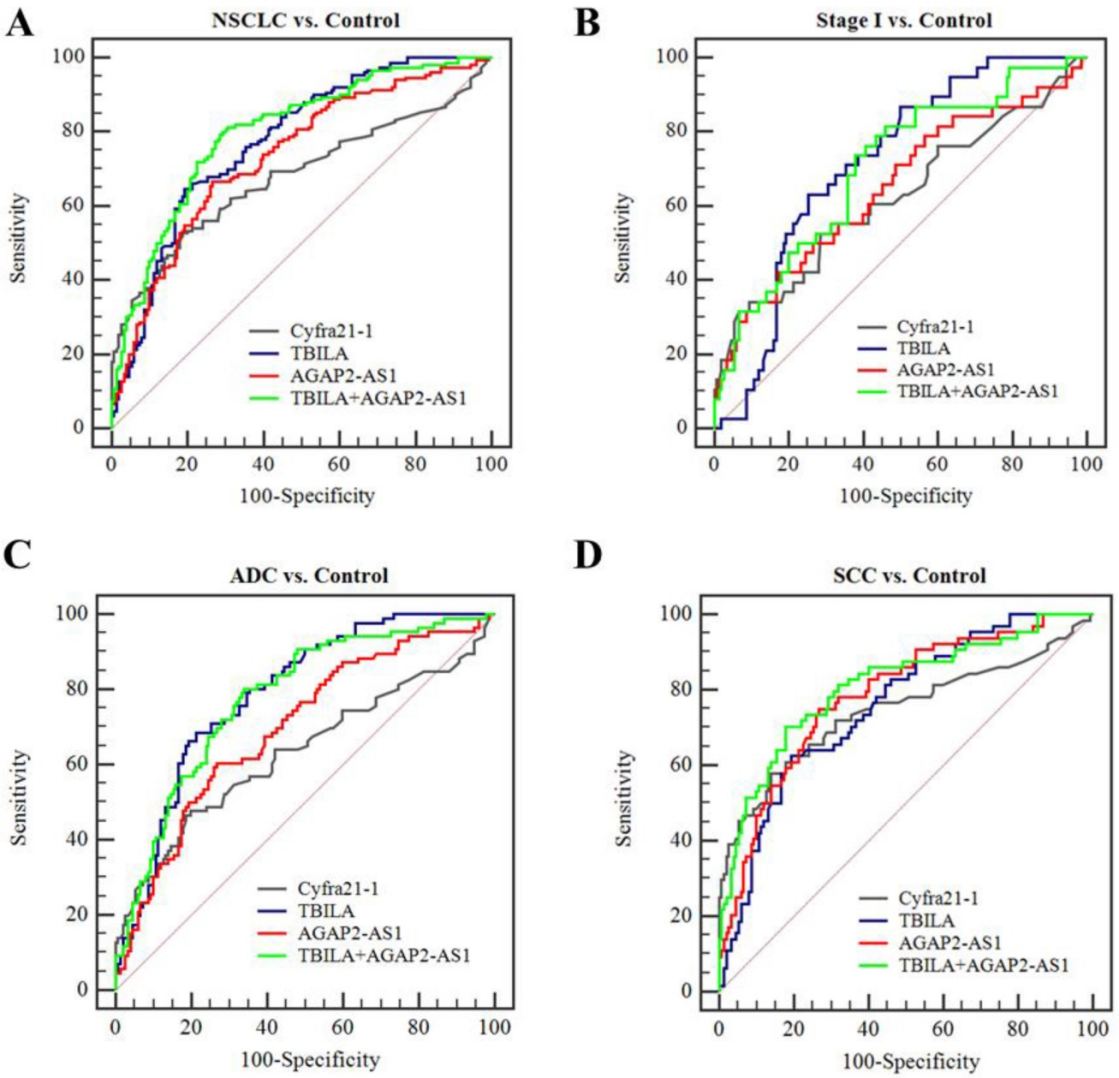
Diagnostic efficiency of individual or combined exosomal lncRNAs for NSCLC.** (A-B)** ROC curve analysis for exosomal lncRNAs in distinguishing NSCLCs overall or stage I NSCLC patients versus healthy controls.** (C-D)** ROC curve analysis for exosomal lncRNAs for ADC patients or SCC patients versus healthy controls.

At present, we assessed the diagnostic efficiency of individual exosomal lncRNA in NSCLC patients. As shown in Fig. 5, TBILA or AGAP2-AS1 alone exhibited high discriminatory capacity for NSCLC patients, as compared to Cyfra21-1. In particular, TBILA exhibited higher discriminatory capacity for all NSCLC patients, stage I NSCLC patients and ADC patients, while AGAP2-AS1 displayed higher AUC in distinguishing SCC patients from healthy controls. On the basis of the ROC curve, we obtained an optimum TBILA cut-off value of 0.923 (sensitivity, 64.7%; specificity, 80.7%) and AGAP2-AS1 cut-off value of 1.12 (sensitivity, 66.7%; specificity, 73.3%) for NSCLC diagnosis, respectively.

**Figure 6:**
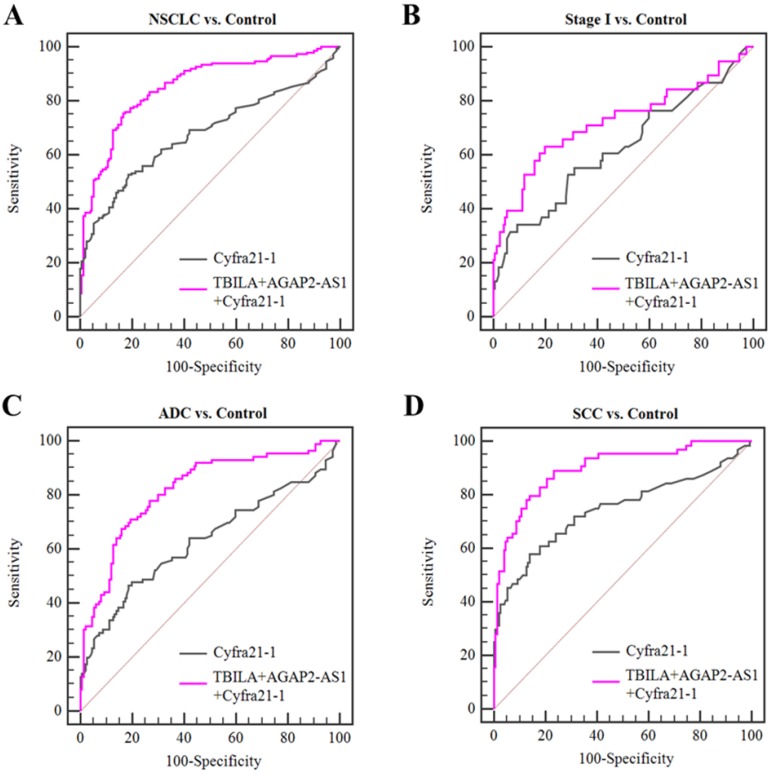
Diagnostic efficiency of the combination of exosomal lncRNAs and serum Cyfra21-1 for NSCLC.** (A-B)** ROC curve analysis for the combination of exosomal lncRNAs and Cyfra21-1 in distinguishing NSCLCs overall or stage I NSCLC patients versus healthy controls. **(C-D)** ROC curve analysis for the combination of exosomal lncRNAs and Cyfra21-1 for ADC patients or SCC patients versus healthy controls.

Based on the aforementioned results, we further assessed the diagnosis value of lncRNAs-based panel using binary logistic regression. The results showed that the combined lncRNAs did not provide better results than individual lncRNAs (Table 5, Fig 5). Furthermore, we combined the two exosomal lncRNAs and serum biomarker Cyfra21-1 and investigated whether this combination could improve the diagnostic capacity for NSCLC. The results demonstrated that the TBILA/AGAP2-AS1/Cyfra21-1 combination displayed a high AUC value (0.853, Table 5, Fig. 6A) in distinguishing all NSCLC patients from healthy controls. The combination provided moderate discriminatory capacity for patients with stage I NSCLC (AUC: 0.723, Table 5, Fig. 6B). In addition, the TBILA/AGAP2-AS1/Cyfra21-1 combination yielded optimal diagnostic efficiency for ADC patients (AUC: 0.815, Table 5, Fig. 6C) and SCC patients (AUC: 0.895, Table 5, Fig. 6D).

Taken together, these results indicated that serum exosomal lncRNA TBILA and AGAP2-AS1 have better diagnostic efficiency for NSCLC patients, and the combination of these exosomal lncRNAs and Cyfra21-1 could improve the diagnostic efficiency.

## Discussion

Exosomes are endosome-derived vesicles that are secreted by almost all types of cells 35, which contains an abundant of cargo that may be used as biomarkers for diseases 36. Recently, emerging evidence have reported that lncRNAs are stable in blood exosomes and have diagnostic potential in cancer management 37. However, the diagnostic value of circulating exosomal lncRNAs in NSCLC remains unclear. Herein, our data demonstrated that serum exosomal lncRNA TBILA and AGAP2-AS1 might be potential diagnostic biomarkers for NSCLC.

In the present study, we first successfully isolated serum exosomes by a polymer precipitation kit, and then identified by TEM, NTA and western blot analysis. So far, there are multiple isolation methods have been developed based on exosomes size, polymer precipitation, immunoaffinity capture, and microfluidics-derived chip 38. Among these methods, ultracentrifugation-based methods remain the gold standard, and polymer precipitation method is one of the commonly used methods due to the easily handled without requiring specialized equipment and fast isolation process. Subsequently, we sought to determine the high level and stable lncRNA in serum from patients with NSCLC. A total of nine candidate lncRNAs, which were differentially expressed in NSCLC tissues and played critical roles in carcinogenesis in the latest literature 26-34, were detected. We demonstrated that three exosomal lncRNAs (TBILA, AGAP2-AS1 and SOX2OT) were significantly elevated in serum from NSCLC patients compared with healthy controls by two independent sets. Furthermore, we also found that three exosomal lncRNAs were increased in the early stage of NSCLC. Recently, Zhang et al. reported that serum exosomal MALAT-1 was highly expressed in NSCLC patients 39. Teng et al identified plasma exosomal SOX2-OT levels significantly upregulated in NSCLC patients 40. In another study, Li et al. found that serum exosomal GAS5 was downregulated in patients with early NSCLC 41. In our experiment, higher amounts of exosomal lncRNAs were also observed in the serum of patients with ADC and SCC but there was no significant difference between tumor subtypes. It suggested that exosomal lncRNA is a universal biomarker in patients with NSCLC.

Given the higher levels of three exosomal lncRNAs (TBILA, AGAP2-AS1 and SOX2OT), we further analyzed the relationship between these exosomal lncRNAs and clinical characteristics of NSCLC patients. Our results showed that TBILA was associated with tumor size, and AGAP2-AS1 was linked to lymph node metastasis and TNM stage. In addition, we demonstrated that the levels of TBILA and AGAP2-AS1 were significantly reduced after surgery, which indicates that their levels are related to the presence of the tumor in NSCLC patients. Unexpectedly, the correlation between exosomal lncRNA SOX2OT levels and clinical characteristics was not observed. It's worth pointing out that high level of plasma exosomal SOX2OT was also found in pancreatic ductal adenocarcinoma 42, perhaps it reduces the specificity as a biomarker in the diagnosis of lung cancer. Thus, we focused on exosomal lncRNA TBILA and AGAP2-AS1 in further study. Considering that stability is an important prerequisite for biomarkers, we next assessed the levels of two exosomal lncRNAs when exosomes suspension treated with RNase A. The results indicated that RNase A treatment did not significantly decrease the levels of exosomal lncRNAs. Further experiments showed that prolonged room temperature exposure also did not influence the levels of serum exosomal lncRNAs. Present results were supported by Skog et al.^43^, who reported there was only less than 7% decrease in exosomal RNA content following RNase treatment. Important previous data also revealed that serum-derived exosomes could protect lncRNA HOTTIP in gastric cancer from prolonged room temperature and multiple freeze-thaw cycles 44. Results from our study are consistent with these previous findings, which indicates that the candidate exosomal lncRNAs are stable in serum exosomes and they are worth to further study.

Finally, we evaluated the diagnostic efficiency of serum exosomal lncRNAs by ROC curve analysis. The results showed that individual TBILA or AGAP2-AS1 exhibited relatively high discriminatory capacity for NSCLCs from healthy subjects. Meanwhile, we obtained an optimum TBILA cut-off value of 0.923 (sensitivity, 64.7%; specificity, 80.7%) and AGAP2-AS1 cut-off value of 1.12 (sensitivity, 66.7%; specificity, 73.3%) for NSCLC diagnosis, respectively. Of note, the final cut-off value for exosomal lncRNAs needs to be further validated in prospective and multicenter studies. Considering that early detection of NSCLC will greatly improve outcome, we next evaluated the diagnostic performance of exosomal lncRNAs for early stage of NSCLC. Here, we showed that the diagnostic sensitivity of exosomal TBILA was 63.2% for early stage patients, which was higher than that of AGAP2-AS1 (42.1%) and Cyfra21-1 (36.3%). In fact, further work is needed to improve the diagnosis efficiency of serum exosomal lncRNAs in the early stage of NSCLC. A recent study has reported a new multi-colour fluorescence digital PCR, compared with the most commonly used qRT-PCR, the new method is more sensitive and can absolutely quantify exosomal lncRNAs from the peripheral blood of patients with lung cancer 45. It has been well known that therapeutic decisions are heavily depended on the pathologic subtype of NSCLC 46, we further explored the diagnostic value of exosomal lncRNAs for different pathologic tumor subtypes. Our data showed that exosomal TBILA exhibited a satisfactory diagnostic capacity for ADCs (AUC, 0.788; sensitivity, 68.6%; specificity, 78.7%), while AGAP2-AS1 showed the highest AUC in the differentiating SCCs from healthy controls (AUC, 0.784; sensitivity, 75.0%; specificity, 73.3%). Furthermore, we assessed the diagnostic value of lncRNAs-based panel for NSCLC patients. Although the combined lncRNAs did not provide better results than individual lncRNA, it is interesting that the combination of two exosomal lncRNAs and Cyfra21-1 could improve the diagnostic accuracy for NSCLC patients. Thus, these data suggest that exosomal lncRNAs TBILA and AGAP2-AS1 might be promising biomarker for the diagnosis of patients with NSCLC, and the combination of two exosomal lncRNAs and Cyfra21-1 might be a good diagnostic panel for NSCLC.

Certainly, we are also aware of potential limitations in our study. Firstly, the sample size was relatively limited, and it is necessary to validate the exosomal lncRNAs in a larger population. Secondly, it will also be requisite to investigate the specificity of these exosomal lncRNAs in the diagnosis of NSCLC by examining it in patients with other cancer types. Moreover, we mainly focused on the diagnostic efficiency of exosomal lncRNAs in this study, the prognostic value of exosomal lncRNAs deserved to be further investigated. Additionally, further researches are worthy to elucidate the function and mechanisms of exosomal lncRNAs in NSCLC.

## Conclusions

**Figure 7:**
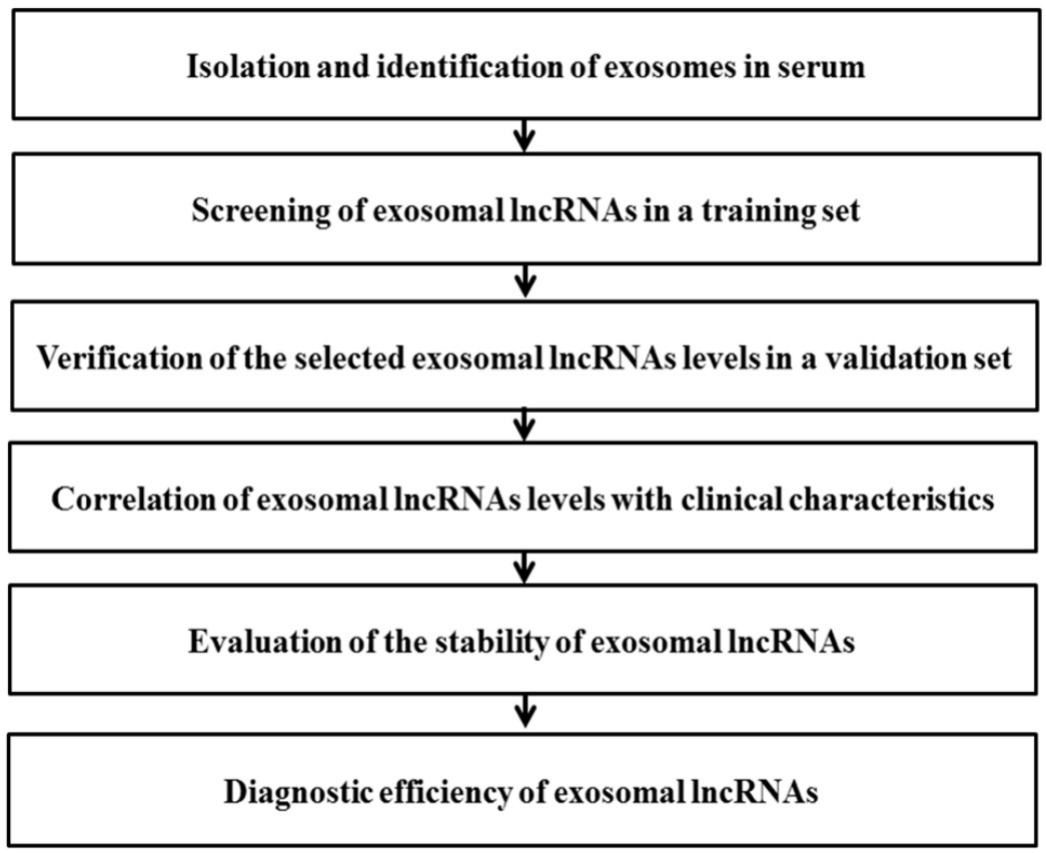
Schematic diagram illustrates the multi-step process of identifying potential exosomal lncRNAs in serum for diagnosis of NSCLC.

In this study, a multi-step process was designed to evaluate potential circulating exosomal lncRNAs for diagnosis of NSCLC (Fig. 7). The data showed that serum exosomal lncRNA TBILA and AGAP2-AS1 are highly expressed in patients with NSCLC. Furthermore, these exosomal lncRNAs have high diagnostic efficiency and appear to be promising biomarkers for diagnosis of NSCLC, which should be confirmed in larger studies.

## Figures and Tables

**Table 1:** Clinical characteristics of study subjects.

Categories	Training set				Validation set		
	Healthy	NSCLC		*p*-value	Healthy	NSCLC	*p*-value
Number	50	50			100	100	
Age (mean ± SD), years	56.3±9.2	58.2±10.2		0.35	58.1±10.7	60.8±10.2	0.41
Gender							
Male	27	31		0.54	60	65	0.56
Female	23	19			40	35	
NSCLC subtype							
ADC		29				57	
SCC		21				43	
Cyfra21-1 (ng/mL)							
≤ 3.3		19				36	
> 3.3		31				64	
Tumor stage							
I		12				26	
II		6				13	
III		15				20	
IV		17				41	
							

Abbreviation: NSCLC, non-small cell lung cancer; ADC, adenocarcinoma; SCC, squamous cell carcinoma; Cyfra21-1, cytokeratin 19 fragment.

**Table 2:** Primer sequences used in qRT-PCR.

Genes	Sequence (5'-3')
*TBILA*	F: 5'-CTGAGAGCCACTTCCACCAT-3' R: 5'-TTTTACACCCTGCCCTGTTG-3'
*AGAP2-AS1*	F: 5'-TACCTTGACCTTGCTGCTCTC-3' R: 5'-TGTCCCTTAATGACCCCATCC-3'
*SOX2OT*	F: 5'-GCTCGTGGCTTAGGAGATTG-3' R: 5'-CTGGCAAAGCATGAGGAACT-3'
*PVT1*	F: 5'-TTGGCACATACAGCCATCAT-3' R: 5'-GCAGTAAAAGGGGAACACCA-3'
*NSCLCAT1*	F: 5'-TGCAAATTGTGGTTCCTGGG-3' R: 5'-CCGAAGAGCAACACACCAAA-3'
*SNHG20*	F: 5'-CCTGTGTGCCTGGAAAGGAAT-3' R: 5'-GGCACAGGAACCACAGAGTAT-3'
*SNHG1*	F: 5'-TAACCTGCTTGGCTCAAAGGG-3' R: 5'-CAGCCTGGAGTGAACACAGA-3'
*Linc00673*	F: 5'-CCGTGTAAAGAGGCCAGTGT-3' R: 5'-ACACGAGCCTTCACCATCAG-3'
*SNHG12*	F: 5'-TCTGGTGATCGAGGACTTCC-3' R: 5'-ACCTCCTCAGTATCACACACT-3'
*GAPDH*	F: 5'-GGCGCTGAGTACGTCGTGGA-3' R: 5'-GTGGTGCAGGAGGCATTGCTGAT-3'

Abbreviations: F stands for forward; R stands for reverse.

**Table 3:** The nine exosomal lncRNAs levels in the training set [median (interquartile range)].

LncRNAs	Healthy controls	NSCLCs	*p*-value
TBILA	0.41 (0.16-0.80)	1.09 (0.73-1.99)	<0.001
AGAP2-AS1	0.95 (0.48-1.72)	1.85 (0.87-3.01)	<0.001
SOX2OT	0.81 (0.39-1.58)	1.80 (0.81-3.75)	<0.001
PVT1	0.83 (0.46-2.03)	1.10 (0.70-2.40)	0.44
NSCLCAT1	1.24 (0.60-2.04)	1.62 (0.72-2.52)	0.47
SNHG20	1.28 (0.96-2.40)	1.48 (0.81-2.50)	0.72
SNHG1	1.76 (0.95-4.00)	1.63 (0.86-2.23)	0.25
Linc00673	1.20 (0.67-2.27)	1.50 (0.61-2.70)	0.31
SNHG12	0.74 (0.42-1.14)	0.53 (0.39-0.86)	0.17

**Table 4:** Correlation between three exosomal lncRNA levels and clinical characteristics of patients with NSCLC (n=150) [median (interquartile range)].

Categories	Cases	TBILA	*p*-value	AGAP2-AS1	*p*-value	SOX2OT	*p*-value
Age (years)							
≤ 60	72	1.09 (0.60-1.67)	0.37	1.35 (0.75-2.75)	0.37	1.14 (0.69-2.10)	0.40
> 60	78	1.12 (0.63-1.99)		1.59 (0.92-2.71)		1.46 (0.79-3.01)	
Gender							
Male	96	1.15 (0.60-1.74)	0.92	1.72 (0.88-2.80)	0.17	1.30 (0.81-2.75)	0.48
Female	54	1.09 (0.56-2.35)		1.17 (0.72-2.09)		1.60 (0.45-2.65)	
Tumor size (cm)							
≤ 3	51	0.74 (0.51-1.73)	0.03	1.32 (0.66-2.34)	0.13	1.50 (0.88-2.75)	0.39
> 3	99	1.17 (0.78-2.25)		1.50 (0.98-3.15)		1.88 (0.78-3.09)	
Lymph node metastasis							
Negative	74	1.11 (0.63-1.79)	0.62	1.23 (0.65-2.22)	0.04	1.22 (0.50-1.82)	0.34
Positive	76	1.12 (0.60-2.06)		1.80 (0.98-3.35)		1.06 (0.64-2.59)	
TNM stage							
I+II	57	1.05 (0.70-1.48)	0.12	1.18 (0.66-1.98)	0.002	1.23 (0.86-1.91)	0.09
III+IV	93	1.30 (0.69-2.13)		1.85 (0.97-3.21)		1.61 (0.70-3.60)	

**Table 5:** The diagnostic efficiency of exosomal lncRNAs and serum Cyfra21-1 in distinguishing NSCLC cases from controls.

Groups	Biomarkers	AUC (95%CI)		SN	SP	*p*-value
NSCLC vs. Control	Cyfra21-1		0.679 (0.623-0.731)	52.7	81.3	<0.001
	TBILA^a^		0.775 (0.723-0.821)	64.7	80.7	<0.001
	AGAP2-AS1^b^		0.734 (0.680-0.783)	66.7	73.3	<0.001
	TBILA+AGAP2-AS1		0.799 (0.749-0.843)	81.3	69.3	<0.001
	TBILA+AGAP2-AS1+Cyfra21-1		0.853 (0.808-0.891)	91.4	80.7	<0.001
Stage I vs. Control	Cyfra21-1		0.625 (0.551-0.694)	36.3	93.3	=0.03
	TBILA		0.715 (0.645-0.779)	63.2	74.7	<0.001
	AGAP2-AS1		0.650 (0.577-0.718)	42.1	82.7	=0.005
	TBILA+AGAP2-AS1		0.704 (0.633-0.768)	73.7	62.0	<0.001
	TBILA+AGAP2-AS1+Cyfra21-1		0.723 (0.654-0.786)	63.2	80.0	<0.001
ADC vs. Control	Cyfra21-1		0.632 (0.567-0.693)	46.5	81.3	=0.001
	TBILA		0.788 (0.730-0.838)	68.6	78.7	<0.001
	AGAP2-AS1		0.696 (0.633-0.754)	60.5	73.3	<0.001
	TBILA+AGAP2-AS1		0.777 (0.719-0.829)	80.2	66.0	<0.001
	TBILA+AGAP2-AS1+Cyfra21-1		0.815 (0.790-0.862)	87.2	66.0	<0.001
SCC vs. Control	Cyfra21-1		0.744 (0.688-0.808)	57.8	86.0	<0.001
	AGAP2-AS1		0.784 (0.723-0.837)	75.0	73.3	<0.001
	TBILA		0.757 (0.694-0.813)	62.5	80.7	<0.001
	TBILA+AGAP2-AS1		0.808 (0.749-0.858)	70.3	82.0	<0.001
	TBILA+AGAP2-AS1+Cyfra21-1		0.895 (0.846-0.933)	81.2	87.3	<0.001
						

Abbreviations: AUC, area under the ROC curve; SN, sensitivity; SP, specificity. ^a^ The diagnostic cut-off value for TBILA was 0.923.^b^ The diagnostic cut-off value for AGAP2-AS1 was 1.12.

## Abbreviations

NSCLC: non-small cell lung cancer
ADC: adenocarcinoma
SCC: squamous cell carcinoma
LDCT: low-dose computerized tomography
Cyfra21-1: cytokeratin 19 fragment
CEA: carcinoembryonic antigen
lncRNAs: long non-coding RNAs
TBILA: TGFβ-induced lncRNA
AGAP2-AS1: AGAP2 antisense RNA 1
TEM: transmission electron microscopy
NTA: nanoparticle tracking analysis
PCR: polymerase chain reaction
F: forward
R: reverse
SD: standard deviation
ROC: receiver operating characteristic curves
AUC: area under the curves
EDS: exosomes-depleted serum
SN: sensitivity
SP: specificity
